# Nafamostat Mesylate for the Hypercoagulable State of SARS-CoV-2 With Renal Replacement Therapy: A Case Report

**DOI:** 10.7759/cureus.52641

**Published:** 2024-01-20

**Authors:** Koki Nakada, Daisuke Hirai, Koichi Seta, Kei Nishiyama

**Affiliations:** 1 Emergency Department, Kyoto Prefectural University of Medicine, Kyoto, JPN; 2 Nephrology Department, National Hospital Organization Kyoto Medical Center, Kyoto, JPN; 3 Emergency and Critical Care Division, Niigata University, Niigata, JPN

**Keywords:** non-fractioned heparin, heparin, critical emergency medicine, renal replacement therapy (rrt), intensive care unit, hypercoagulation, nafamostat, continuous renal replacement therapy (crrt), thrombosis, covid-19

## Abstract

Being a dialysis patient is one of the risks for severe coronavirus disease 2019 (COVID-19) cases. In addition, there have been many reports of coagulation abnormalities in severe COVID-19 cases; these also make dialysis management more difficult. In this study, we report a case of severe COVID-19 in a hemodialysis patient who had coagulation in the dialysis circuit with unfractionated heparin (UFH), which could be managed without intracircuit obstruction when nafamostat mesylate (NM) was used in combination with unfractionated heparin.

## Introduction

A hypercoagulable state and the development of thrombotic circuit occlusion requiring circuit replacement are reported to make renal replacement therapy (RRT) difficult to perform in patients with severe acute respiratory syndrome coronavirus 2 (SARS-CoV-2) [1]. However, unfractionated heparin (UFH) is not highly effective against coagulation abnormalities of coronavirus disease 2019 (COVID-19) [2,3].

Nafamostat mesylate (NM) is a serine protease inhibitor that potently inhibits proteolytic enzymes such as thrombin, plasmin, and trypsin. It has been used for the treatment of pancreatitis and coagulation abnormalities, as well as anticoagulation for RRT in Japan, and NM has been shown to be a potential new option for COVID-19 treatment because of its antiviral activity [4,5].

We report our experience in COVID-19 of a case of thrombotic circuit occlusion on RRT that was difficult to address with UFH but could be avoided with NM.

## Case presentation

**Figure 1:**
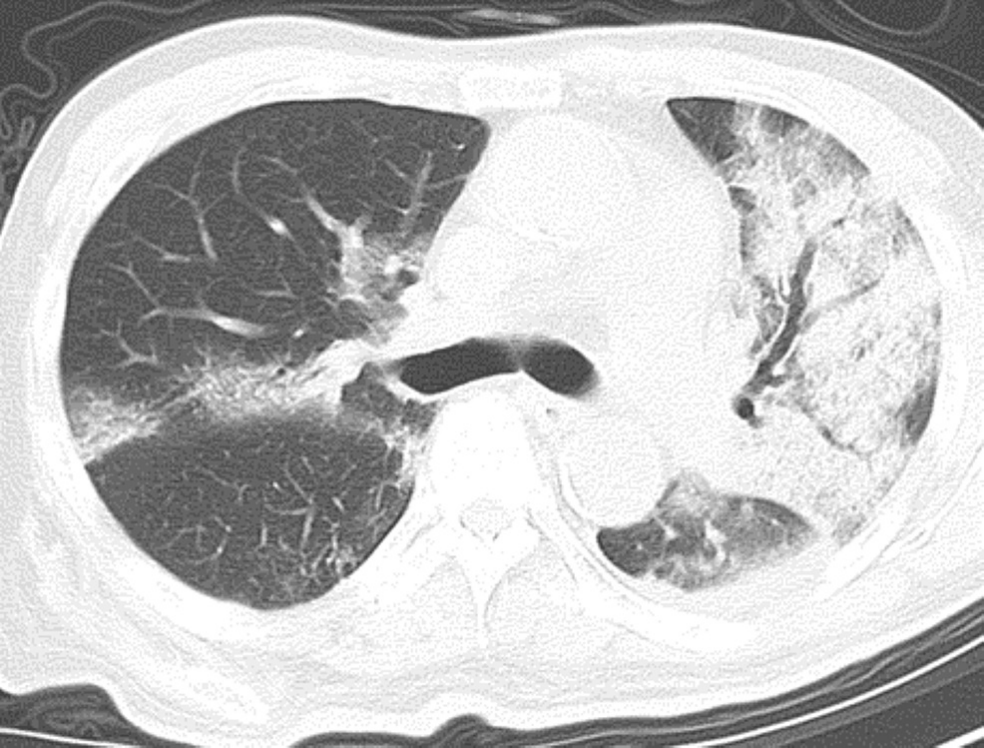
The unenhanced computed tomography (CT) scan that was taken three days before admission at another hospital The CT scan showed multiple patchy ground glass opacity in the subpleural area of both lungs and consolidation and air bronchogram sign on the left upper lobe of the lung

A 72-year-old male chronic maintenance dialysis patient was transferred to our hospital with refractory pneumonia in April 2020. He had preexisting hypertension and diabetes mellitus. He had been treated with antimicrobial agents at another hospital as an outpatient for bacterial pneumonia with fever and cough for the past 10 days. His respiratory failure worsened, and a computed tomography (CT) scan revealed severe pneumonia, so he was transferred to our emergency department. The polymerase chain reaction (PCR) test was positive for SARS-CoV-2, so he was diagnosed with COVID-19 and treated in the ICU (Figure 1).

**Figure 2:**
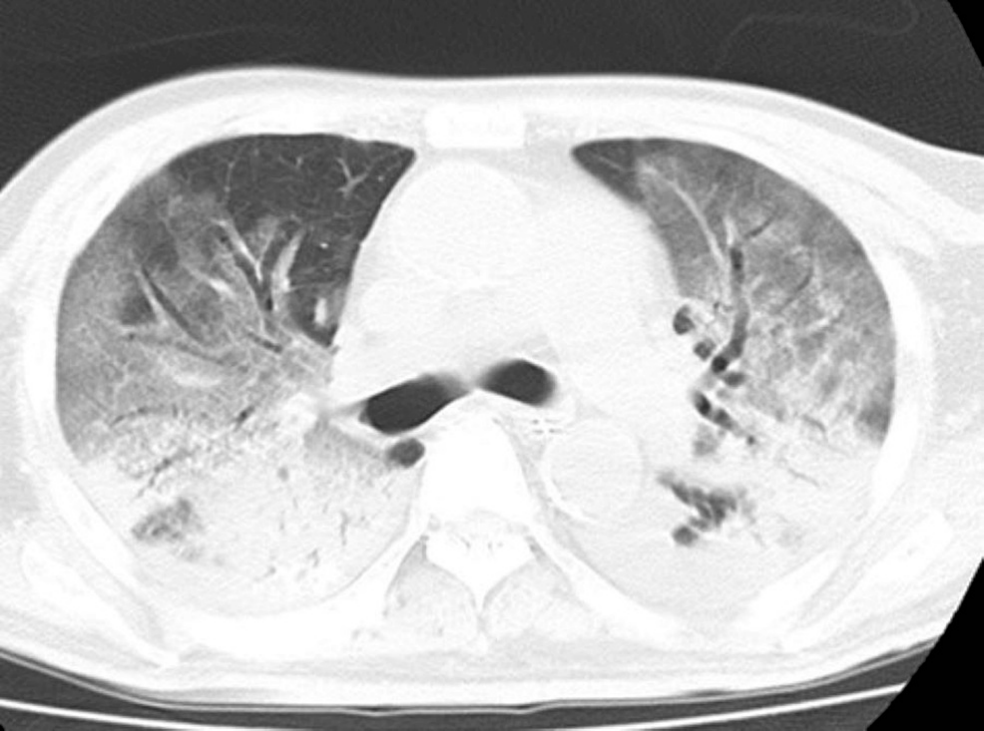
Follow-up unenhanced CT on day 10 The CT scan showed an increase in ground glass opacity density in both lungs CT: computed tomography

Due to the worsening respiratory condition on hospital day 3, he was intubated, and ventilator management was started. Favipiravir treatment was started on hospital day 4. A rapid increase in the D-dimer was observed from hospital day 9, and a rapid decrease in the partial pressure of oxygen (PaO_2_)/fraction of inspired oxygen (FiO_2_) ratio was observed on hospital day 10, so CT imaging was performed again, which showed an exacerbation of bilateral slit-glass shadows, and lung protective management was initiated as acute respiratory distress syndrome (ARDS) (Figure 2).

Anticoagulant therapy was started on hospital day 12 as prophylaxis for deep vein thrombosis. UFH was started using activated partial thromboplastin time (APTT) as an indicator, which was administered continuously to maintain APTT at 1.5-2 times the baseline. He showed a drop in antithrombin III level, so we had to replenish it frequently. A venous thrombus was found in the right femoral vein on ultrasound on hospital day 19 . On hospital day 27, he suffered a shock state, and a contrast-enhanced CT showed pulmonary artery embolism. The patient developed multiple organ failure and died on hospital day 39.

Thrombotic circuit occlusion in RRT

We started RRT on hospital day 3 in the ICU, and the thrombotic circuit occlusion was resistant to UFH and difficult to manage despite increasing doses of UFH (the time to thrombotic occlusion of the circuit was 185, 110, 240, 180, and 150 minutes). However, the thrombotic circuit occlusion did not occur after the anticoagulant was changed to NM (35 mg/kg) on hospital days 31 and 34 (Table 1).

**Table 1:** Renal replacement therapy (RRT), concomitant anticoagulation, laboratory results, and thrombotic circuit blockage APTT, activated partial thromboplastin time; AT, antithrombin; ECUM, extracorporeal ultrafiltration; HD, hemodialysis; NM, ‎nafamostat mesylate; N/A, not available; PT, prothrombin time; SLED, sustained low-efficiency dialysis; UFH, unfractionated heparin

Day	RRT	Anticoagulation before RRT	Anticoagulation during RRT	PT (seconds)	APTT (seconds)	D-dimer (μg/mL)	AT III (%)	Platelet	Time to thrombotic occlusion of the circuit (minutes)
3	HD	None	UFH: 500 IU bolus, 250 IU/hour	12.6	43.6	N/A	73	135000	No occlusion
6	HD	None	UFH: 750 IU bolus, 500 IU/hour	N/A	N/A	N/A	N/A	N/A	No occlusion
11	SLED	None	UFH: 500 IU bolus, 250 IU/hour	14.9	52.1	54.8	51	101000	185
13	SLED＋ECUM	8000 IU/day	UFH: 750 IU bolus, 500 IU/hour	19.5	71.4	53.5	34	73000	No occlusion
17	HD	7000 IU/day	UFH: 250 IU bolus, 500 IU/hour	14.0	51.8	15.3	45	68000	110
20	SLED	9000 IU/day	UFH: 500 IU bolus, 750 IU/hour	12.5	54.0	17.9	36	94000	240
25	HD	12000 IU/day	UFH: 250 IU bolus, 750 IU/hour	14.7	64.1	14.7	27	78000	180
28	HD	12000 IU/day	UFH: 250 IU bolus, 500 IU/hour	17.7	62.8	12.2	30	87000	150
31	SLED+ECUM	10000 IU/day	NM: 35 mg/hour	19.4	71.9	8.4	30	51000	No occlusion
34	SLED+ECUM	10000 IU/day	NM: 35 mg/hour	15.5	58.2	20.2	18	54000	No occlusion

## Discussion

Coagulation abnormalities in COVID-19 have been implicated in significant microvascular thrombosis associated with widespread alveolar and interstitial inflammation with a similar mechanism to macrophage activation syndrome [6], and despite prophylactic anticoagulation, 9.5% of patients were reported to have thrombosis complications [7]. Furthermore, there was a significantly higher frequency of thrombotic complications in COVID-19 patients with ARDS, as well as a higher incidence of intrapathway coagulation on RRT [8].

UFH has been recommended for the treatment of coagulopathy in COVID-19, but its efficacy is not clear [2,9]. If the mechanism of coagulopathy in COVID-19 is different from that of the usual coagulopathy, a different approach may need to be considered. In this case, despite the fact that UFH was able to maintain sufficient APTT prolongation in the present case, it failed to control intracircuit coagulation during RRT; however, the thrombotic circuit occlusion did not occur after the anticoagulant was changed to NM. This fact suggests that NM can be effective as a treatment for the hypercoagulable state of SARS-CoV-2 (Appendices).

There may be some limitations. We experienced this case in April 2020, so we had to manage with the very first guideline of COVID-19. Now, we can choose several medications for COVID-19 treatment [10], so we may have better results even with UFH. We just started using NM on days 31 and 34, and the patient expired on day 39. So, we might not get enough to monitor the patient's status, coagulation effects over a period of time, and adverse effects.

## Conclusions

We experienced a case with thrombotic circuit occlusion on RRT that was difficult to address with UFH but could be avoided with NM. COVID-19 antithrombotic therapy with NM may prevent venous thrombus and pulmonary artery embolism. NM could be an effective treatment modality for the hypercoagulable state caused by SARS-CoV-2.

## Appendices

Table 2 shows the respiratory condition and laboratory results of the patient.

**Table 2:** Detail of respiratory condition and laboratory results APTT, activated partial thromboplastin time; AT, antithrombin; Fib, fibrinogen; FDP, fibrin/fibrinogen degradation products; N/A, not available; PCT, procalcitonin; PT, prothrombin time; P/F, partial pressure of oxygen (PaO_2_)/fraction of inspired oxygen (FiO_2_)

Day	P/F ratio	D-dimer (μg/mL)	AT III (%)	PT (seconds)	Platelet (×1000/μL)	Fib (×10 mg/dL)	APTT (seconds)	FDP (mg/dL)	PCT
1	170	3	83	11	140	44	38	N/A	N/A
2	N/A	N/A	74	11	129	42	44	11	N/A
3	N/A	N/A	73	13	135	39	44	12	N/A
4	213	3	84	13	142	51	48	N/A	18
5	208	5	84	14	135	48	45	N/A	16
6	204	N/A	N/A	N/A	125	N/A	N/A	N/A	N/A
7	232	5	69	15	115	48	44	N/A	9
8	210	6	64	15	109	55	46	N/A	N/A
9	200	18	64	15	122	53	42	N/A	7
10	100	28	55	16	115	47	44	N/A	6
11	160	55	51	15	101	38	52	N/A	N/A
12	170	50	40	17	80	34	59	169	13
13	170	53	34	20	73	33	71	98	N/A
14	196	35	35	21	66	29	67	N/A	11
15	237	14	38	19	79	33	55	19	N/A
16	219	8	40	19	77	37	56	11	8
17	N/A	15	45	14	68	37	52	20	N/A
18	172	11	50	14	78	41	56	18	8
19	165	12	42	13	84	40	52	17	N/A
20	140	18	36	13	94	38	54	N/A	N/A
21	150	22	35	12	71	34	49	N/A	11
22	154	24	52	13	58	31	54	N/A	10
23	163	24	40	13	67	31	54	N/A	N/A
24	170	14	44	13	64	25	57	N/A	12
25	205	15	27	15	78	22	64	N/A	N/A
26	200	13	25	15	69	18	60	N/A	18
27	150	13	24	15	67	15	59	N/A	N/A
28	120	12	30	18	87	15	63	19	16
29	128	13	15	21	72	14	75	N/A	N/A
30	155	12	15	19	68	13	63	N/A	21
31	160	8	30	19	51	11	72	N/A	15
32	167	13	18	17	54	13	60	N/A	N/A
33	180	18	18	16	49	13	58	N/A	9
34	150	20	18	16	54	16	58	23	N/A
35	150	21	45	16	62	19	54	N/A	N/A
36	132	23	38	15	68	22	51	N/A	N/A
37	146	23	31	15	65	23	53	N/A	7
38	107	24	33	15	126	30	64	N/A	N/A
39	N/A	41	30	30	127	26	120	N/A	N/A
